# Effect of Sleep-Disordered Breathing on Exercise Capacity after Myocardial Infarction — A Cross-Sectional Study

**DOI:** 10.31083/j.rcm2410299

**Published:** 2023-10-20

**Authors:** Danuta Loboda, Michalina Stepanik, Jacek Durmala, Rafal Gardas, Krzysztof S. Golba

**Affiliations:** ^1^Department of Electrocardiology and Heart Failure, Medical University of Silesia in Katowice, 40-635 Katowice, Poland; ^2^Department of Rehabilitation, Medical University of Silesia in Katowice, 40-635 Katowice, Poland

**Keywords:** cardiac rehabilitation, exercise capacity, myocardial infarction, obstructive sleep apnea, sleep-disordered breathing

## Abstract

**Background:**

Exercise capacity reflects the cardiovascular risk after myocardial infarction (MI). The study aims to evaluate the impact of sleep-disordered breathing (SDB) on exercise capacity after MI.

**Methods:**

Consecutive patients referring to outpatient cardiac rehabilitation up to 28 days after MI and participating in the Polish Managed Care after Acute Myocardial Infarction (MC-AMI) program were included. On admission, we assessed the presence and the severity of SDB using the home sleep apnea test (HSAT), patients’ maximum exercise capacity on a treadmill exercise stress test (EST), and a 6-minute walk test (6MWT), as well as the effect of SDB on the results obtained. In the multivariate analysis, we verified the strength of the observed associations concerning age, anthropometric parameters, and left ventricular ejection fraction (LVEF).

**Results:**

A total of 254 patients aged 60.00 (interquartile range 51.00–67.00), including 39 (15.4%) women, with technically adequate HSAT, constituted the study group. Mild SDB was found in 82 (32.3%), moderate in 54 (21.3%), and severe in 51 (20.1%) patients. Among those diagnosed with SDB, obstructive sleep apnea (OSA) was dominant in 167 (89.8%). With the worsening of SDB, the distance in 6MWT and the maximum physical exertion achieved in EST, expressed in metabolic equivalents (METs) and maximal heart rate (MHR), decreased. The linear regression analysis confirmed the following: (1) inversely proportional relationship between the respiratory event index and METs, MHR, and 6MWT distance (*p* = 0.005, *p* = 0.008, and *p* = 0.004), and the maximum apnea duration and MET and 6MWT distance (*p* = 0.042 and *p* = 0.002); and (2) directly proportional relationship between mean arterial oxygen saturation (${\mathrm{SpO}}_{2}$) during sleep and MET, MHR, and 6MWT distance (*p* = 0.019, *p* = 0.006, and *p* = 0.013), and minimum ${\mathrm{SpO}}_{2}$ and MET and MHR (*p* = 0.040 and *p* < 0.001). However, the independent risk factors for impaired exercise capacity, determined using multivariable regression analysis, were age, female sex, higher body mass index (BMI), and decreased LVEF, but not SDB parameters.

**Conclusions:**

SDB negatively impacts exercise capacity after MI. However, the strength of this association may be less pronounced due to the interaction of risk factors common for SDB and impaired exercise capacity, e.g., sex, age, BMI, and LVEF.

## 1. Introduction

Exercise capacity reflects general fitness and body health and depends on the functions of the cardiovascular (CV), respiratory, and musculoskeletal systems [1]. Low physical activity and cardiorespiratory fitness (CRF) are strong and independent predictors of CV diseases, coronary artery events, and all-cause or CV mortality in the general population [1, 2, 3, 4]. Importantly, in patients with CV diseases, including coronary artery disease (CAD), the maximum exercise capacity achieved by cardiopulmonary exercise testing (CPET) typifies the risk of all-cause mortality [1, 5]. Exercise capacity is affected by age, sex, body mass index (BMI), and some comorbidities [6, 7, 8]. One of them is sleep-disordered breathing (SDB), predominantly obstructive sleep apnea (OSA) [9, 10, 11]. Poorer exercise capacity measured in metabolic equivalents (METs) is also a predictor of increased all-cause mortality in patients with SDB, particularly in the presence of CAD [12].

SDB is common in patients with CAD [13, 14, 15, 16], including cardiac rehabilitation (CR) participants [17, 18, 19]. SDB, a chronic disorder, consists of repeated apnea and hypopnea during sleep, with subsequent desaturation and cardiovascular autonomic imbalance [20, 21]. The disorder is accompanied by oxidative stress, chronic inflammation, insulin resistance, endothelial dysfunction, platelet aggregation abnormalities, arterial stiffness, resistant hypertension (HA), and increased atherosclerotic CV risk [15, 20, 21]. SDB was recognized as a risk factor for increased mortality in CAD patients [22, 23]. The presence of advanced OSA after myocardial infarction (MI) promotes left ventricular (LV) remodeling with LV dilatation and the development of heart failure (HF) with reduced LV ejection fraction (HFrEF) [24]. In turn, resistant HA causes LV hypertrophy with subsequent HF with preserved LV ejection fraction and atrial fibrillation (AF) episodes [25, 26]. Both factors limit the exercise capacity of post-MI patients. Moreover, some studies suggest an abnormal CV exercise response in SDB patients, including chronotropic incompetence [9, 27, 28] and inadequate increase in blood pressure (BP) [27, 28, 29, 30]. Another cardiac impairment factor may be nocturnal hypoxia [31]. Furthermore, skeletal muscle structure and function may likely be altered in SDB patients with chronic severe hypoxia and harm performance and exercise tolerance [32]. SDB is more common in older people with multimorbidity who are overweight or obese, which can further impair CRF [33, 34]. Sleep fragmentation also leads to excessive daytime sleepiness, concentration and memory deterioration, fatigue, tiredness, and lack of energy, possibly worsening compliance with CV pharmacotherapy, lifestyle changes, and physical activity after MI [35, 36]. However, whether SDB has a significant effect on exercise capacity in patients after MI remains unclear.

The study aims to evaluate the impact of SDB on the initial exercise capacity of patients referred for cardiac rehabilitation after MI.

## 2. Materials and Methods

### 2.1 Study Group

We evaluated consecutive patients referred for CR and hospitalized in the Day Treatment Cardiac Rehabilitation Department of the Upper-Silesian Medical Center (Katowice, Poland). The inclusion criteria for the study were a history of MI (up to 28 days before admission) and participation in the Polish Cardiac Society, the National Health Fund, and the Ministry of Health’s comprehensive Managed Care after Acute Myocardial Infarction (MC-AMI, KOS-ZAWAL) program [37]. The MC-AMI program provides early CR and one-year extensive cardiac care to patients diagnosed with MI, regardless of its type (ST-elevation MI or non-ST-elevation MI) and the treatment method in the acute period (procedural or conservative). According to the program’s rules, outpatient CR does not include patients with HF with significantly reduced LV ejection fraction (LVEF), especially below 35%, or in Functional Class IV in New York Heart Association (NYHA) and patients needing overnight care. In addition, patients with incomplete coronary revascularization and low coronary flow reserve by treadmill exercise stress test (EST) or with signs and symptoms of circulatory decompensation (i.e., pulmonary congestion or severe peripheral edema) were excluded from CR. They were referred for retreatment in the cardiology department. Moreover, the exclusion criteria for the study included the current treatment of SDB with positive airway pressure (PAP) or intraoral devices.

### 2.2 Assumptions of the Study

We used anthropometric and echocardiographic measurements and data concerning comorbidities from medical records collected upon admission to the rehabilitation department.

Then we assessed the presence and severity of SDB in a studied cohort with a portable polysomnography system (Alice NightOne, Philips Respironics) used as a Home Sleep Apnea Test (HSAT) [38]. The HSAT recordings were analyzed manually following the recommendations of the American Academy of Sleep Medicine [38]. We defined sleep apnea as a 90%–100% reduction in airflow through the airway for at least 10 s. Episodes of apnea with preserved breathing movements were classified as obstructive, while those without breathing movements were classified as central. We noted hypopnea when there was a decrease in the airflow by 30% that lasted ≥10 s and led to a ≥4% decrease in oxygen saturation of hemoglobin (${\mathrm{SpO}}_{2}$). We assessed the severity of SDB using the respiratory event index (REI), defined as the frequency of apneas or hypopneas per hour of recording (events/h). The REI less than 5 we described as correct. We classified SDB with an REI of 5–14 events/h as mild, 15–30 events/h as moderate, and >30 events/h as severe.

Next, we estimated the patients’ maximum exercise capacity on a treadmill EST with electrocardiography and BP monitoring (according to the modified Bruce protocol) [39] and a six-minute walk test (6MWT) [40]. We presented exercise capacity as an estimated amount of oxygen consumed in METs, maximum heart rate (MHR), and its percentage adjusted to age (MHR%) obtained in EST and as a distance in meters (m) in a 6MWT. Then we assessed the effect of SDB on the results obtained. In the multivariate analysis, we verified the strength of the observed associations concerning age, anthropometric parameters, and LVEF.

### 2.3 Statistical Analysis

The results were analyzed using MedCalc Version 22.006 (MedCalc Software Ltd., Ostend, Belgium). The quantitative parameters were presented as the median and interquartile range (IQR). Qualitative data were expressed as numbers and percentages. The normality of the distribution of the variables was assessed with the Kolmogorov-Smirnov test. Differences in the frequency and size of anthropometric and HSAT parameters, maximum exercise capacity parameters, LVEF, and coexisting diseases by SDB severity were calculated using the chi-square test with the Cochran–Armitage test for trend or the Kruskal–Wallis test with the Conover *post-hoc* analysis and the Jonckheere–Terpstra test for trend. Independent risk factors for impaired exercise capacity were determined using univariable and multivariable regression analysis, with candidate variables of age, male sex, body weight, BMI, LVEF, REI, average and maximal apnea episode duration, average and minimal ${\mathrm{SpO}}_{2}$ during sleep, and the percentage of total sleep time with oxyhemoglobin saturation below 90% (TST90). In multivariable regression analysis, according to the stepwise method, a variable was entered into the model if its associated significance level was <0.05 and removed from the model if its associated significance level was >0.10. A *p*-value less than 0.05 was considered statistically significant.

## 3. Results

Initially, 264 Caucasians aged >18 years, who participated in CR after MI, were included in the study. The technically adequate HSAT was performed on 254 patients aged 60.00 (IQR 51.00–67.00), including 39 (15.4%) women—these constituted the study group.

SDB was found in 187 (73.6%) patients in the study group: mild in 82 (32.3%), moderate in 54 (21.3%), and severe in 51 (20.1%). Moderate and severe SDB were more common in men than women, *p *= 0.031. Among the SDB patients, OSA was dominant in 167 (89.8%), and central sleep apnea (CSA) in 19 (10.2%).

SDB severity tended to increase with age, BMI, and neck circumference, with a significant difference in BMI and neck circumference between the subgroup without SDB and those with severe SDB in a head-to-head comparison (Table 1). However, patients with severe SDB did not differ in comorbidities except for incidences of diabetes (trend) (Table 2). CSA was more common in patients with HFrEF (8 [36.4%]) than others (12 [7.2%]), *p <* 0.001. Of the patients enrolled, 90.0% used beta-blockers, 94.0% angiotensin-converting enzyme inhibitors (ACE-Is) or angiotensin receptor blockers (ARBs), and 27.7% aldosterone receptor antagonists (MRAs). The mean BP (the average of several initial measurements taken during CR) was within the normal range (i.e., <140/90 mmHg) in 190 [77.6%] participants. The remaining patients required pharmacotherapy optimization, and the BP values were within the range of 140–159/90–99 mmHg in 44 (18.0%), 160–179/100–109 mmHg in 8 (3.3%), and ≥180/110 mmHg in 3 (1.2%). BP control did not differ according to SDB severity, *p *= 0.968.

**Table 1.** **Characteristics of anthropometric parameters per sleep-disordered breathing severity**.

Predictor	All participants	SDB severity				*p*-value for trend
		None	Mild	Moderate	Severe	
		(I)	(II)	(III)	(IV)	
no (%)	254	67 (26.4)	82 (32.3)	54 (21.3)	51 (20.1)	
Age (years), M (IQR)	60.00 (51.00–67.00)	58.00 (47.00–65.75)	59.50 (52.00–66.00)	59.50 (53.00–67.00)	62.00 (53.00–69.00)	0.017
Sex (male), no (%)	215 (84.6)	51 (76.1)	69 (84.1)	50 (92.6)	45 (88.2)	0.027
Body mass (kg), M (IQR)	87.60 (78.00–98.00)	84.20 (73.35–96.60)	88.15 (78.00–98.70)	88.75 (78.50–98.00)	91.00 (79.95–102.33)	0.016
BMI (${\mathrm{kg/m}}^{2}$), M (IQR)	28.91 (26.31–32.10)	28.20 (25.42–30.30)	28.65 (26.10–33.00)	29.35 (26.70–31.95)	30.00 (26.93–34.94)	0.004
						I ≠ IV
Neck (cm), M (IQR)	42.00 (40.00–44.00)	41.00 (38.25–43.00)	42.00 (40.00–44.00)	43.00 (40.50–45.00)	43.00 (41.00–44.38)	<0.001
						I ≠ II, I ≠ III, I ≠ IV

The results are given for all cases and for each of the subgroups. BMI, body mass index; M, median; Neck, neck circumference; no, number; IQR, interquartile range; SDB, sleep-disordered breathing; ≠ means *p*-value < 0.05 in the Conover post-hock analysis.

**Table 2.** **Characteristics of coexisting diseases per sleep-disordered breathing severity**.

Predictor	All participants	SDB severity				*p*-value for trend
		None	Mild	Moderate	Severe	
No (%)	254	67 (26.4)	82 (32.3)	54 (21.3)	51 (20.1)	
MI, no (%)	254 (100.0)	67 (100.0)	82 (100.0)	54 (100.0)	51 (100.0)	>0.999
Hypertension, no (%)	208 (81.9)	50 (74.6)	69 (84.1)	43 (79.6)	46 (90.2)	0.066
HFmrEF, no (%)	48 (18.9)	9 (13.4)	20 (24.4)	11 (20.4)	8 (15.7)	0.058
HFrEF, no (%)	24 (9.4)	3 (4.5)	8 (9.8)	5 (9.3)	8 (15.7)	0.392
AF, no (%)	16 (6.3)	4 (6.0)	4 (4.9)	3 (5.6)	5 (9.8)	0.415
Stroke, no (%)	7 (2.8)	1 (1.5)	1 (1.2)	3 (5.6)	2 (3.9)	0.206
Diabetes, no (%)	56 (22.0)	10 (14.9)	17 (20.7)	14 (25.9)	15 (29.4)	0.043
CKD, no (%)	16 (6.9)	7 (10.9)	2 (2.6)	3 (6.2)	4 (9.1)	0.819
${\mathrm{Hypercholesterolemia}}^{1}$, no (%)	161 (69.1)	47 (73.4)	50 (64.9)	30 (62.5)	34 (77.3)	0.868
COPD, no (%)	12 (4.7)	3 (4.5)	4 (4.9)	5 (9.3)	0 (0.0)	0.544
Osteoarthritis, no (%)	17 (7.3)	3 (4.7)	10 (13.0)	0 (0.0)	4 (9.1)	0.952
Smoking ${\mathrm{status}}^{2}$, no (%)	83 (32.7)	29 (43.3)	24 (29.3)	17 (31.5)	13 (25.5)	0.061

The results are given for all cases and for each of the subgroups. ^1^ total cholesterol >5.0 mmol/L or lipid-lowering treatment; ^2^ Smokers or former smokers (up to five years); AF, atrial fibrillation; CKD, chronic kidney disease with an estimated glomerular filtration rate of <60 mL/min/1.73 $m^{2}$; COPD, chronic obstructive pulmonary disease; HFmrEF, heart failure with mildly reduced ejection fraction of 41–49%; HFrEF, heart failure with reduced left ventricular ejection fraction of ≤40%; MI, myocardial infarction; no, number; SDB, sleep-disordered breathing.

Table 3 reflects changes in HSAT parameters per SDB severity. The differences between subgroups, especially without or mild SDB, and those with severe SDB were significant for most parameters. The evaluation of trends confirmed the worsening of respiratory parameters with increasing SDB severity based on REI. Table 4 presents the characteristics of the maximum exercise capacity parameters and LVEF per SDB severity. As SDB worsened, the 6MWT distance and the maximum physical exertion achieved in EST, expressed in METs and MHR, tended to decrease. The 6MWT, METs, and MHR median values differed between the subgroup of patients with severe SDB and the others. However, there were no differences in the MHR% achieved. In addition, as the SDB severity increased, so did the degree of fatigue/dyspnea assessed on Borg’s scale.

**Table 3.** **Characteristics of home sleep apnea test parameters per sleep-disordered breathing severity**.

Predictor	All participants	SDB severity				*p*-value for trend
		None	Mild	Moderate	Severe	
		(I)	(II)	(III)	(IV)	
No (%)	254	67 (26.4)	82 (32.3)	54 (21.3)	51 (20.1)	
REI (events/hour), M (IQR)	11.25 (4.90–25.40)	2.30 (1.53–3.78)	9.10 (6.90–11.40)	20.25 (17.90–25.30)	41.90 (33.93–50.35)	<0.001
						I ≠ II, I ≠ III, I ≠ IV
Participants with OSA/CSA, no (%)	168 (66.1)/19 (7.5)	–	76 (92.7)/6 (7.3)	48 (88.9)/6 (11.1)	44 (86.3)/7 (13.7)	0.225
Average episode duration (s), M (IQR)	21.90 (18.20–25.70)	18.80 (15.10–25.48)	22.90 (19.50–26.60)	21.30 (19.30–24.50)	22.70 (19.38–25.38)	0.009
						I ≠ II, I ≠ IV
Max episode duration (s), M (IQR)	54.50 (42.00–74.00)	36.50 (22.75–55.75)	57.50 (43.38–73.63)	54.75 (46.00–76.50)	65.00 (56.00–88.38)	<0.001
						I ≠ II, I ≠ III, I ≠ IV, II ≠ IV, III ≠ IV
Average ${\mathrm{SpO}}_{2}$ (%), M (IQR)	93.00 (92.00–94.00)	93.00 (92.00–95.00)	93.00 (92.00–94.00)	93.00 (92.00–94.00)	92.50 (91.00–94.00)	0.003
						I ≠ IV, II ≠ IV, III ≠ IV
Minimal ${\mathrm{SpO}}_{2}$ (%), M (IQR)	85.00 (82.00–88.00)	88.00 (84.00–90.00)	85.00 (83.00–88.00)	84.00 (82.00–87.00)	82.00 (78.00–85.00)	<0.001
						I ≠ II, I ≠ III, I ≠ IV, II ≠ IV
TST90 (%), M (IQR)	1.57 (0.10–7.19)	0.30 (0.00–2.83)	1.00 (0.07–3.20)	2.80 (0.47–9.09)	7.19 (1.73–25.74)	<0.001
						I ≠ III, I ≠ IV, II ≠ III, II ≠ IV, III ≠ IV
Sleepiness on ESS (pts), M (IQR)	5.0 (3.0–8.0)	4.0 (2.3–7.0)	5.0 (3.0–9.0)	5.0 (4.0–9.0)	4.0 (3.0–8.0)	0.177

The results are given for all cases and for each of the subgroups. CSA, central sleep apnea; ESS, Epworth Sleepiness Scale; M, median; no, number; OSA, obstructive sleep apnea; IQR, interquartile range; REI, respiratory event index; SDB, sleep-disordered breathing; ${\mathrm{SpO}}_{2}$, arterial oxygen saturation estimated by pulse oximetry, TST90, the percentage of total sleep time spent with oxyhemoglobin saturation below 90%; ≠ means *p*-value < 0.05 in the Conover post-hock analysis.

**Table 4.** **Characteristics of the maximum exercise capacity parameters and left ventricular ejection fraction per sleep-disordered breathing severity**.

Predictor	All participants	SDB severity				*p*-value for trend
		None	Mild	Moderate	Severe	
		(I)	(II)	(III)	(IV)	
No (%)	254	67 (26.4)	82 (32.3)	54 (21.3)	51 (20.1)	
LVEF (%), M (IQR)	55.00 (48.00–55.00)	55.00 (50.00–55.00)	52.00 (48.00–55.00)	55.00 (48.00–55.00)	55.00 (48.00–55.00)	0.579
NYHA class, M (IQR)	2.0 (1.0–2.0)	2.0 (1.0–2.0)	2.0 (1.0–2.0)	1.5 (1.0–2.0)	2.0 (1.0–2.0)	0.165
6MWT (m), M (IQR)	645.00 (518.00–814.00)	666.50 (525.50–867.50)	657.00 (564.00–806.00)	668.50 (496.50–860.00)	552.00 (457.00–686.50)	0.021
						I ≠ IV, II ≠ IV, III ≠ IV
Metabolic equivalents, M (IQR)	7.15 (6.10–9.50)	7.20 (6.33–9.50)	7.50 (6.90–9.50)	7.60 (5.23–9.50)	6.80 (5.60–7.90)	0.015
						I ≠ IV, II ≠ IV, III ≠ IV
MHR (bpm), M (IQR)	119.50 (108.00–132.50)	127.00 (112.25–137.00)	120.00 (110.25–133.00)	117.00 (104.50–129.00)	116.50 (103.00–128.00)	0.003
						I ≠ III, I ≠ IV
MHR% (%), M (IQR)	77.00 (69.00–82.00)	79.00 (70.00–82.75)	76.00 (71.00–82.00)	73.50 (67.00–81.50)	77.00 (69.00–83.00)	0.41
Fatigue level (pts), M (IQR)	13.0 (14.0–16.0)	12.0 (9.0–14.0)	13.0 (10.0–14.0)	13.0 (10.0–14.0)	14.0 (10.0–14.0)	0.035

The results are given for all cases and for each of the subgroups. bpm, heart beats per minute; LVEF, left ventricular ejection fraction; M, median; no., number; NYHA, New York Heart Association; MHR, maximal heart rate; MHR%, proportion of the age-based predictions of the maximal heart rate; IQR, interquartile range; 6MWT, six-minute walk test; no., number; SDB, sleep-disordered breathing; ≠ means *p*-value < 0.05 in the Conover post-hock analysis. Fatigue level is given in Borg’s rate of perceived exertion scale.

**Fig. 1.**
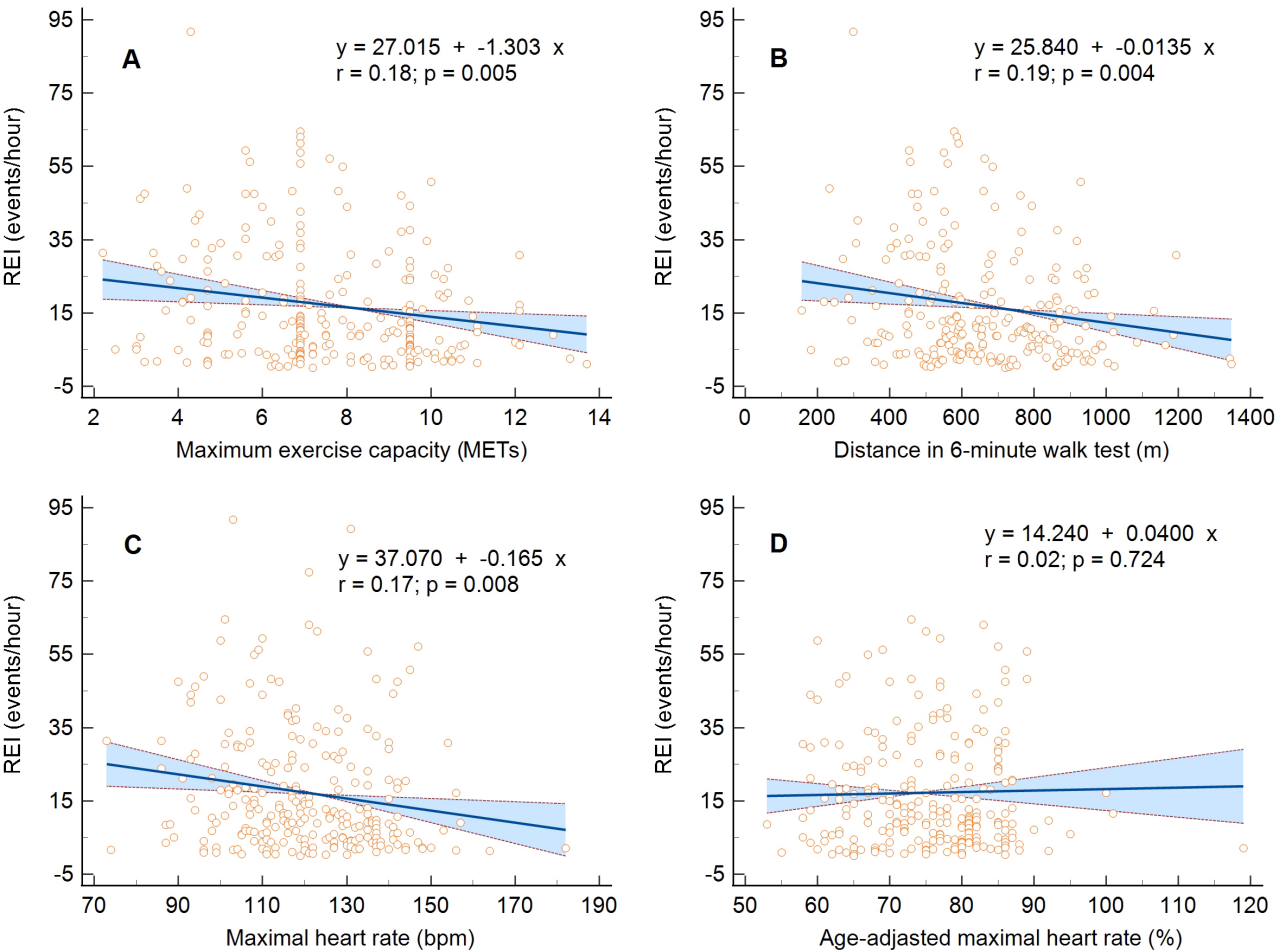
**Linear regression and 95% confidence interval for the relationship between maximum exercise capacity and the respiratory event index.** (A) Metabolic equivalents, (B) maximal heart rate, (C) distance in six-minute walk test, and (D) percentage of age-adjusted maximal heart rate. MET, metabolic equivalents; REI, respiratory event index.

**Fig. 2.**
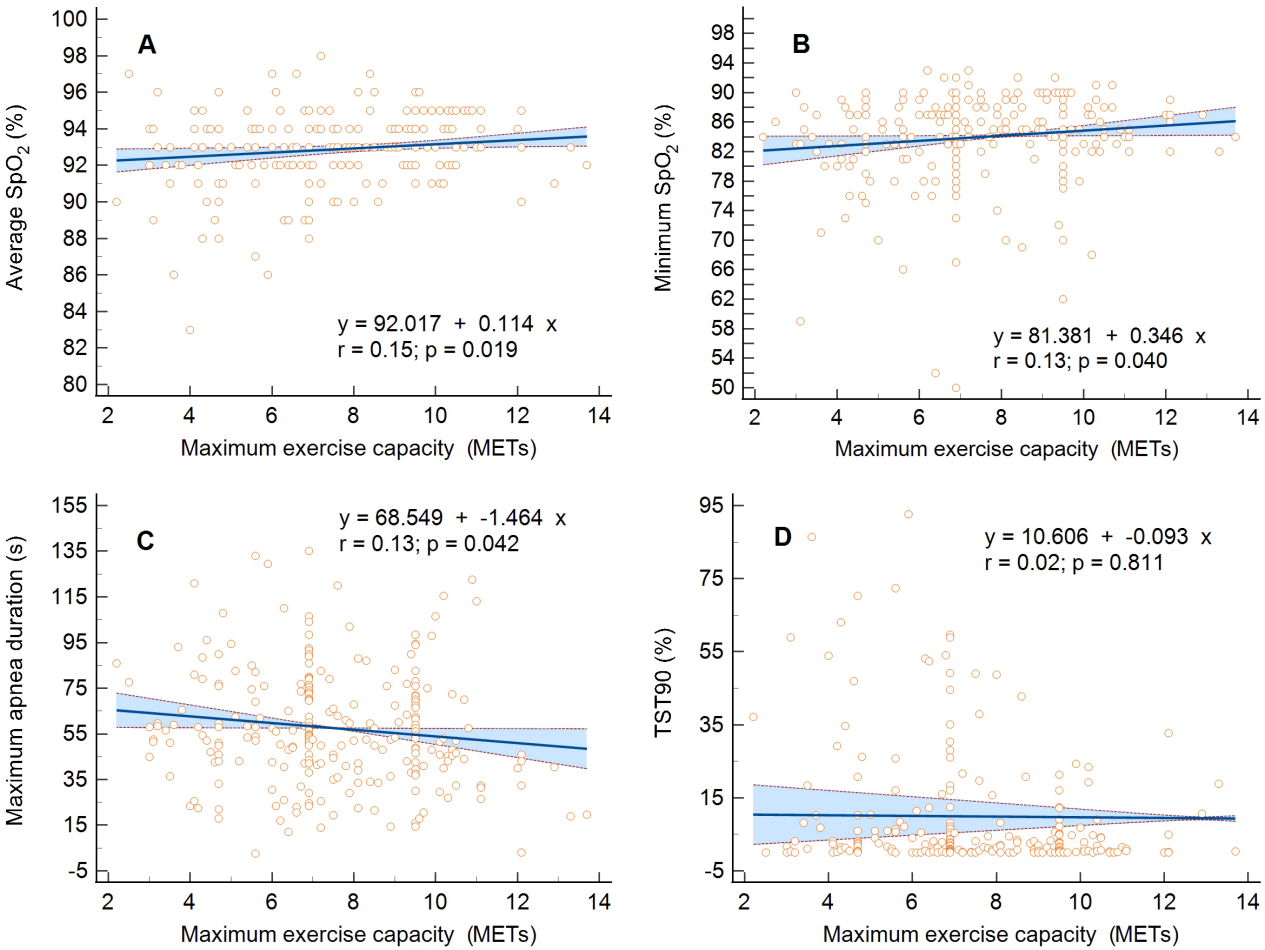
**Linear regression and 95% confidence interval for the relationship between maximum exercise capacity expressed in metabolic equivalents (METs) and the respiratory parameters.** (A) Average oxygen saturation of hemoglobin (${\mathrm{SpO}}_{2}$), (B) minimum oxygen saturation of hemoglobin, (C) maximum sleep apnea duration, and (D) the percentage of total sleep time spent with oxyhemoglobin saturation below 90% (TST90).

The linear regression analysis confirmed the inversely proportional relationship between the REI and the maximum exercise capacity expressed only in METs, MHR, and 6MWT distance, not MHR% (Fig. 1A–D). Moreover, there was a proportional relationship between mean ${\mathrm{SpO}}_{2}$ and METs, MHR, or 6MWT distance (*p *= 0.019, *p *= 0.006, and *p *= 0.013, respectively) and minimum ${\mathrm{SpO}}_{2}$ and METs or MHR (*p *= 0.040 and *p <* 0.001) and an inversely proportional relationship between maximum apnea duration and METs or 6MWT distance (*p *= 0.042 and *p *= 0.002), (Fig. 2A–D). The hypoxic burden assessed as the time spent with desaturation <90% (TST90) did not affect the maximum exercise capacity assessed in METs (*p *= 0.811) and 6MWT distance (*p *= 0.061), only those in MHR (*p <* 0.001). In addition, maximum exercise capacity in METs per EST and meters per 6MWT was inversely related to age, BMI, and LVEF. The independent risk factors for impaired exercise capacity, determined using a multivariable regression analysis, were age, sex, BMI, and LVEF, not SDB severity (Table 5).

**Table 5.** **Risk factors for impaired exercise capacity expressed in metabolic equivalents, maximal heart rate, and distance in a six-minute walk test in univariable and multivariable regression analysis**.

Predictor	Maximum exercise capacity expressed in metabolic equivalents					
	Full model			Stepwise regression		
	Coefficient	Standard error	*p*-value	Coefficient	Standard error	*p*-value
Age (years)	–2.106	0.267	<0.001	–0.087	0.011	<0.001
Sex (male)	-	-	-	1.030	0.316	0.001
Body weight (kg)	–0.792	0.425	0.064	not included in the model		
BMI (${\mathrm{kg/m}}^{2}$)	–0.621	0.114	<0.001	–0.124	0.024	<0.001
LVEF (%)	0.866	0.172	<0.001	0.105	0.016	<0.001
REI (events/hour)	–1.303	0.457	0.005	did not enter the model		
Average episode duration (s)	–0.128	0.156	0.450	not included in the model		
Maximal episode duration (s)	–1.464	0.715	0.042	did not enter the model		
Average ${\mathrm{SpO}}_{2}$ (%)	0.114	0.048	0.019	did not enter the model		
Minimal ${\mathrm{SpO}}_{2}$ (%)	0.346	0.168	0.040	did not enter the model		
TST90 (%)	–0.093	0.386	0.811	not included in the model		
Predictor	Exercise capacity expressed as maximal heart rate					
	Full model			Stepwise regression		
	Coefficient	Standard error	*p*-value	Coefficient	Standard error	*p*-value
Age (years)	–0.264	0.036	<0.001	–0.684	0.091	<0.001
Sex (male)	-	-	-	did not enter the model		
Body weight (kg)	–0.053	0.058	0.357	not included in the model		
BMI (${\mathrm{kg/m}}^{2}$)	–0.048	0.017	0.006	–0.732	0.205	<0.001
LVEF (%)	0.064	0.027	0.018	0.256	0.128	0.046
REI (events/hour)	–0.165	0.061	0.008	did not enter the model		
Average episode duration (s)	–0.029	0.021	0.168	not included in the model		
Maximal episode duration (s)	–0.144	0.096	0.135	not included in the model		
Average ${\mathrm{SpO}}_{2}$ (%)	0.019	0.007	0.006	did not enter the model		
Minimal ${\mathrm{SpO}}_{2}$ (%)	0.062	0.017	<0.001	did not enter the model		
TST90 (%)	–0.133	0.027	<0.001	did not enter the model		
Predictor	Exercise capacity expressed as a distance in a six-minute walk test					
	Full model			Stepwise regression		
	Coefficient	Standard error	*p*-value	Coefficient	Standard error	*p*-value
Age (years)	–0.022	0.003	<0.001	–9.281	1.099	<0.001
Sex (male)	-	-	-	103.257	32.267	0.002
Body weight (kg)	–0.008	0.004	0.049	not included in the model		
BMI (${\mathrm{kg/m}}^{2}$)	–0.005	0.001	<0.001	–12.468	2.396	<0.001
LVEF (%)	0.006	0.002	0.001	7.505	1.917	<0.001
REI (events/hour)	–0.013	0.005	0.004	did not enter the model		
Average episode duration (s)	–0.002	0.002	0.174	did not enter the model		
Maximal episode duration (s)	–0.024	0.008	0.002	did not enter the model		
Average ${\mathrm{SpO}}_{2}$ (%)	0.001	0.0005	0.013	not included in the model		
Minimal ${\mathrm{SpO}}_{2}$ (%)	0.003	0.002	0.050	did not enter the model		
TST90 (%)	0.006	0.003	0.061	not included in the model		

BMI, body mass index; LVEF, left ventricular ejection fraction; REI, respiratory event index; ${\mathrm{SpO}}_{2}$, arterial oxygen saturation estimated by pulse oximetry; TST90, the percentage of total sleep time spent with oxyhemoglobin saturation below 90%.

## 4. Discussion

In the study, we assessed the effect of SDB on the maximum exercise capacity of patients from the high CV risk group, i.e., in the early period after MI, referred for CR. Given the impact of CRF on long-term prognosis and mortality in patients with CV diseases, we aimed to assess whether the effect of SDB on exercise capacity in post-MI patients should be considered an independent prognostic factor. We measured maximum exercise capacity as routinely obtained during CR (i.e., estimated oxygen consumption in METs and MHR from treadmill EST or a distance in 6MWT) and portable polysomnography parameters used as HSAT. We evaluated a representative group of patients with mild to severe SDB, 89.8% of whom had OSA. We found a linear relationship between SDB parameters, such as REI, mean and minimum ${\mathrm{SpO}}_{2}$, or maximum apnea time, and maximum exercise capacity assessed above, confirming that SDB negatively affected exercise capacity in patients after MI. However, the strength of this association may be more difficult to prove due to the strong interaction of risk factors common for SDB and impaired exercise capacity, such as sex, older age, higher BMI, and left ventricular systolic dysfunction.

### 4.1 Effect of Exercise Capacity on Long-Term Prognosis in Patients with CAD

Impaired CRF is a risk factor for morbidity, CV complications, and death in patients with CV diseases [1, 5]. On the other hand, among CAD patients, the risk of death from any cause decreases for every MET increase in maximum exercise capacity (hazard ratio 0.83, 95% confidence interval [CI] 0.76–0.91) [5]. Better physical performance is also independently associated with fewer risk factors for CAD and a lower risk of adverse CV events [41, 42]. Therefore, all agents influencing CRF, physical activity, and lifestyle changes in post-MI patients may be relevant as potential targets for medical intervention [43]. The intervention documented to improve CRF in this group is early CR [41, 42, 44, 45, 46]. Exercise improvement of ≥2 METs during CR is associated with a decrease in major adverse cardiac events, CV-related hospitalizations, and unplanned coronary angiography [45]. Moreover, CR may improve the quality of life and reduce anxiety and depression in patients after MI [41, 47].

The assessment of exercise capacity is an integral part of a patient’s examination upon admission to CR [48]. The cut-off point for the minimum CRF level considered normal and associated with lower CV risk is, for men and women, approximately nine and seven METs for 40-year-olds, eight and six METs for 50-year-olds, and seven and five METs for 60-year-olds [2]. In our cohort of 60-year-old men, CV response to exercise was preserved within a range of values associated with lower CV risk (i.e., 7.15 [6.10–9.50] METs). However, in patients with severe SDB, this value was outside the normal range (i.e., 6.80 [5.60–7.90] METs), which may indicate a higher risk in this subgroup. It was documented that, with the coexistence of CAD, the CV risk associated with OSA further increases [22, 23], and impaired exercise capacity in patients with OSA and CAD has a more serious prognostic value and is associated with an increased risk of all-cause mortality [12].

### 4.2 Effect of SDB on Exercise Capacity

Some studies indicate that SDB, predominantly OSA, affects CRF [9, 18, 28, 29, 33, 49, 50, 51]. We also found a significant relationship between SDB severity and maximum exercise capacity in our cohort of post-MI patients. However, CFR also depends on other factors than SDB itself [6, 7, 8], although often identical to SDB risk factors [19, 20], such as sex, age, overweight/obesity, altered body composition (with increased visceral and neck adiposity), diabetes, hypertension, and congestive HF. It is known that maximum exercise capacity is approximately 2 METs lower in women than in men [6] and decreases by 0.1 MET for every year of aging [7]. Generalized loss of muscle mass and obesity also reduce physical performance [8]. In our group, age, and BMI strongly correlated with the severity of the SDB. The prevalence of diabetes also correlated with an increase in the SDB severity. The NYHA class and LVEF did not differ in groups depending on the SDB severity, but the peak exercise performance in METs and the distance in 6MWT was directly proportional to LVEF. Thus, independent risk factors for exercise capacity impairment assessed by multivariate regression analysis were female sex, older age, higher BMI, and decreased LVEF, but not SDB severity. Similar conclusions were drawn by other researchers [10, 12, 52, 53], stating that among patients with OSA, the influence of sex, obesity/BMI, and comorbidities on physical performance may outweigh that of SDB.

It can be assumed that the potential and independent effect of SDB on CRF may be more pronounced in middle-aged patients with severe SDB but without significant comorbidities. Under such conditions, SDB may be the only exercise limiting factor. For other CV diseases, such as AF, ventricular arrhythmias, or HA, the adverse effect of SDB is the most significant in middle-aged cohorts under 60–65 years old [54, 55, 56]. In younger patients, due to the short duration of the disease, the lack of deep nocturnal desaturation, and higher daily physical activity, SDB may not yet cause complications or lead to symptomatic decreases in CRF [11, 57]. Conversely, for the elderly, multiple comorbidities, including HF, lung diseases, and diabetes, as well as the effects of age, obesity, and physical deconditioning, may outweigh the impact of SDB [10, 50].

Powell *et al*. [57] evaluated a group of military personnel whose members were approximately 41 years old and had moderate to severe OSA (an apnea-hypopnea index [AHI] of 32.7 events/h). In the group, the CRF and BP responses were correct, although the MHR trended toward a blunted response. Arora *et al*. [58] described male military personnel who were ten years older and mature in their careers. These individuals had a mean AHI of 29.0 events/h. Their oxygen consumption (${\mathrm{VO}}_{2}$ peak) at peak exercise capacity was already reduced by 8% compared to the control group but remained in the supernormal range. De Carvalho *et al*. [28] found that in middle-aged women (approximately 55 years old), OSA (AHI >5 events/h) reveals its effect on exercise capacity only in the presence of obesity. Importantly, the impact of OSA and obesity on CRF worsening was synergistic (odds ratio 9.40, 95% CI 3.79–23.3 in obese women with OSA vs. 2.88, 95% CI 1.02–8.11 in obese women without OSA). In a group of 50-year-old individuals with moderate to severe OSA and good physical health, Beitler *et al*. [49] found that each 1-unit increase in log-transformed AHI was associated with a decrease in predicted peak oxygen uptake of 3.20% (95% CI 0.53–5.88). In this study, patients with OSA had the same BMI as the participants in the control group but were older. Mansukhani *et al*. [29] revealed a negative dose–response relationship between AHI and METs achieved at EST for a large cohort of obese men (mean BMI 32.4 ${\mathrm{kg/m}}^{2}$) aged approximately 56. As in our cohort, patients with SDB were older and more obese than those without SDB. However, after adjusting for confounding factors, AHI as a continuous variable remained negatively related to exercise capacity. Sonners *et al*. [18] showed 11.3% lower METs in 60-year-old patients with untreated OSA (AHI >5 events/h) referred to CR. The effect was significant after adjusting for sex, although the age and BMI differences appear not to be accounted for in the CRF pre-CR multivariable analysis. A study of elderly (around 70 years) hypertensives by Barbosa *et al*. [51] showed that moderate OSA (mean AHI 24.4 events/h) did not affect peak workloads. On the contrary, in a study by Vitacca *et al*. [33], conducted in patients over 60 years of age but with severe OSA (AHI 38.3 events/h), 29.2% of participants had mild exercise limitation during 6MWT (6MWT below 85% predicted and above the lower limit of normal) and severe up to 31.9% (6MWT below the lower limit of normal). However, in the group with severe OSA, the burden of nocturnal hypoxia (TST90 of 34%) and multimorbidity was high, which may have influenced the outcome. A study by Ben Saad *et al*. [50], carried out in patients with baseline severe OSA (AHI 62 events/h) treated with PAP, revealed the influence of age, anthropometric factors, and comorbidities on CRF. In the group successfully treated for OSA, the distance in 6MWT remained shorter compared to the control group (512 m vs. 585 m, percentage predicted of 83% vs. 100%) and rode on the presence of CV diseases, BMI, first-second-forced-expiratory-volume, and average ${\mathrm{SpO}}_{2}$ during sleep. It was also shorter in older patients.

### 4.3 Effect of SDB on CV Autonomic Regulation and BP Control

Some authors point to chronotropic insufficiency and lower post-exercise heart rate recovery in OSA patients [27, 28, 51]. In addition, researchers describe dysregulation of BP response to exercise with an excessive peak or postexercise BP in SDB cohorts [27, 28, 30]. We refrained from analyzing the chronotropic competence of the sinus node and the exercise BP response because of the extensive use of pharmacotherapy in post-MI patients, including maximum tolerated doses of beta-blockers and ACE-Is, ARBs, or MRAs, which affect MHR and BP control. However, we observed the decrease in MHR proportional to SDB worsening, expressed as REI, mean, or maximal ${\mathrm{SpO}}_{2}$, and hypoxia burden described as TST90.

### 4.4 The Role of SDB Diagnostics in Patients Referred for CR

Regardless of the impact of SDB on CRF, OSA diagnosis in patients referred to CR may be relevant due to the increased likelihood of exercise-related complications and adverse events, such as AF, exercise-related ventricular arrhythmias, and nocturnal atrioventricular blocks [15, 21, 55, 56, 59]. In the era of home-based hybrid cardiac telerehabilitation [46], without direct medical supervision during exercise, the diagnosis of OSA as a risk factor for CV complications may change the patient’s qualification to the CR type. In addition, starting treatment of severe OSA with PAP before CR may contribute to the increase in the patient’s spontaneous physical activity [60], or CRF [18, 61], and an improvement of compliance, quality of life [36], and long-term CV outcome [62].

### 4.5 Limitations of the Study

Women are poorly represented in the study group due to the lower incidence of CAD and MI among women compared to men. Another limitation is using EST and 6MWT to assess maximum exercise capacity instead of the gold standard, i.e., CTEP [63]. However, EST and 6MWT are the standard tests for qualification for the CR model routinely used in practice, and estimated CRF derived from the peak work rate is accepted as an expression of fitness by scientific societies [1, 48]. We used HSAT to diagnose the SDB’s presence and severity rather than level 1 polysomnography [38], as it is easy to use and possible to perform on an outpatient basis; it is also recommended as screening in CR [64]. Moreover, we repeated or rejected all inconclusive or technically inappropriate HSAT results to ensure the credibility of the research. We also did not have the opportunity to analyze respiratory parameters during exercise. However, the available data show that SDB does not limit ventilatory response and gas exchange during peak exercise, even in patients with high AHI and deep desaturation during sleep [65].

## 5. Conclusions

In post-MI patients, SDB negatively impacts the maximum exercise capacity assessed in METs and the distance in 6MWT. However, the strength of this association may be less pronounced due to the strong interaction of risk factors common for SDB and impaired exercise capacity, such as sex, older age, higher BMI, and left ventricular systolic dysfunction.

## Data Availability

The data presented in this study are available on request from the Department of Electrocardiology and Heart Failure, Medical University of Silesia in Katowice (Poland). The data are not publicly available due to privacy restrictions.

## Abbreviations

AF, atrial fibrillation; AHI, apnea-hypopnea index; BMI, body mass index; BP, blood pressure; CAD, coronary artery disease; CPET, cardiopulmonary exercise testing; CR, cardiac rehabilitation; CRF, cardiorespiratory fitness; CV, cardiovascular; CSA, central sleep apnea; EST, exercise stress test, HFmrEF, heart failure with mildly reduced ejection fraction; HFrEF, heart failure with reduced left ventricular ejection fraction; HSAT, home sleep apnea test; LVEF, left ventricular ejection fraction; MC-AMI, Managed Care after Acute Myocardial Infarction program; MHR, maximal heart rate; MHR%, proportion of the age-based predictions of the maximal heart rate; MI, myocardial infarction; NYHA, New York Heart Association; OSA, obstructive sleep apnea; REI, respiratory event index; SDB, sleep-disordered breathing; ${\mathrm{SpO}}_{2}$, arterial oxygen saturation estimated by pulse oximetry; TST90, the percentage of total sleep time spent with oxyhemoglobin saturation below 90%; 6MWT, six-minute walk test.
